# Neighborhood socioeconomic disadvantage is associated with multimorbidity in a geographically-defined community

**DOI:** 10.1186/s12889-019-8123-0

**Published:** 2020-01-06

**Authors:** Alanna M. Chamberlain, Lila J. Finney Rutten, Patrick M. Wilson, Chun Fan, Cynthia M. Boyd, Debra J. Jacobson, Walter A. Rocca, Jennifer L. St. Sauver

**Affiliations:** 1 Department of Health Sciences Research, Mayo Clinic, 200 First Street SW, Rochester, MN 55905 USA; 2 Robert D. and Patricia E. Kern Center for the Science of Health Care Delivery, Mayo Clinic, 200 First Street SW, Rochester, MN 55905 USA; 3 Division of Geriatric Medicine and Gerontology, Johns Hopkins University, 5505 Eastern Avenue, Baltimore, MD 21224 USA; 4 Department of Neurology, Mayo Clinic, 200 First Street SW, Rochester, MN 55905 USA

**Keywords:** Multimorbidity, Socioeconomic status, Area deprivation index, Geocoding, US Census

## Abstract

**Background:**

Persons with low socioeconomic status may be disproportionately at risk for multimorbidity.

**Methods:**

Adults aged ≥20 years on 4/1/2015 from 7 counties in Minnesota were identified using the Rochester Epidemiology Project (population-based sample). A composite measure of neighborhood socioeconomic disadvantage, the area deprivation index (ADI), was estimated at the census block group level (*n* = 251). The prevalence of 21 chronic conditions was obtained to calculate the proportion of persons with multimorbidity (≥2 chronic conditions) and severe multimorbidity (≥5 chronic conditions). Hierarchical logistic regression was used to estimate the association of ADI with multimorbidity and severe multimorbidity using odds ratios (OR).

**Results:**

Among 198,941 persons (46.7% male, 30.6% aged ≥60 years), the age- and sex-standardized (to the United States 2010 census) median prevalence (Q1, Q3) was 23.4% (21.3%, 25.9%) for multimorbidity and 4.8% (4.0%, 5.7%) for severe multimorbidity. Compared with persons in the lowest quintile of ADI, persons in the highest quintile had a 50% increased risk of multimorbidity (OR 1.50, 95% CI 1.39–1.62) and a 67% increased risk of severe multimorbidity (OR 1.67, 95% CI 1.51–1.86) after adjusting for age, sex, race, and ethnicity. Associations were stronger after further adjustment for individual level of education; persons in the highest quintile had a 78% increased risk of multimorbidity (OR 1.78, 95% CI 1.62–1.96) and a 92% increased risk of severe multimorbidity (OR 1.92, 95% CI 1.72–2.13). There was evidence of interactions between ADI and age, between ADI and sex, and between ADI and education. After age 70 years, no difference in the risk of multimorbidity was observed across quintiles of ADI. The pattern of increasing multimorbidity with increasing ADI was more pronounced in women. Finally, there was less variability across quintiles of ADI for the most highly educated group.

**Conclusions:**

Higher ADI was associated with increased risk of multimorbidity, and the associations were strengthened after adjustment for individual level of education, suggesting that neighborhood context plays a role in health above and beyond individual measures of socioeconomic status. Furthermore, associations were more pronounced in younger persons and women, highlighting the importance of interventions to prevent chronic conditions in younger women, in particular.

## Key messages

- In this cross-sectional study including 198,941 persons aged ≥20 years from a 7-county region in Southern Minnesota, increasing prevalence of multimorbidity was observed with increasing area deprivation. Compared with persons in the lowest quintile of area deprivation, persons in the highest quintile had a 50% increased risk of multimorbidity.
- Associations of increasing prevalence of multimorbidity with increasing area deprivation strengthened after adjustment for individual level of education, suggesting that neighborhood context may influence health above and beyond the effect of individual measures of socioeconomic status.
- Interactions were observed between ADI and age, between ADI and sex, and between ADI and individual level of education. In particular, associations were stronger in younger persons and in women.
- Our findings underscore the importance of identifying socioeconomic disparities and implementing early interventions to prevent chronic conditions in younger persons, and in particular in younger women.

## Background

Approximately 1 of 4 adults in the United States have multimorbidity (defined as the presence of 2 or more chronic conditions) and the prevalence of multimorbidity increases with age [1–4]. In the nearly 31 million persons continuously enrolled in fee-for-service coverage in Medicare Parts A and B during 2008 (83.5% ≥65 years of age), the prevalence of multimorbidity was 62% in those aged 65–74 years and 82% in those aged 85 years and older [5]. With the progressive demographic aging of the population and the improved survival for many chronic conditions, the number of individuals with multimorbidity will likely increase over time, resulting in increased costs and burden on the health care system.

The prevalence of multimorbidity varies across populations due in part to differences in sociodemographic factors, such as age and sex [3, 6, 7]. Socioeconomic status is another source of this variation in prevalence of multimorbidity, with higher prevalence of multimorbidity generally observed in persons of lower socioeconomic status [3, 7, 8]. Most studies assessing the relationship of socioeconomic status with multimorbidity focused on individual measures of socioeconomic status, most commonly education and income. Lower education, in particular, was shown to be associated with a greater than 50% increased risk of multimorbidity in a meta-analysis including 10 studies [8]. However, measuring individual socioeconomic variables in large populations is challenging because the data are often not comprehensively captured in electronic medical records. Thus, some studies have assessed the associations between neighborhood measures of socioeconomic status and multimorbidity, utilizing publically available data [8–12]. However, the majority of these studies were conducted in Europe.

To our knowledge, only one prior study was conducted in the United States; however, only housing characteristics were used to measure socioeconomic status. In addition, although adults of all ages were included, differences in the associations by age were not reported [11]. Furthermore, neighborhood context may be independently associated with health above and beyond the effect of individual measures of socioeconomic status [13]. Thus, the purpose of our study was to investigate whether an area-level measure of socioeconomic status, the area deprivation index (ADI), is associated with multimorbidity, whether the association persists after adjustment for an individual measure of socioeconomic status, and whether the association differs by age or sex in a 7-county region in Southern Minnesota.

## Methods

### Study population

This study was conducted using the expanded Rochester Epidemiology Project (E-REP) medical records-linkage system [14]. For this study, we utilized a 7-county high capture region of the E-REP, which captures data from 93.8% of the population in the following counties in Southern Minnesota: Olmsted, Wabasha, Dodge, Mower, Steele, Waseca, and Freeborn. All adults who resided in the 7-county region aged ≥20 years of age on April 1, 2015 and who provided authorization to use their medical records for research (Minnesota Research Authorization; 92% of the REP population) were included in this study (*n* = 206,849). This study was approved by the Mayo Clinic and Olmsted Medical Center Institutional Review Boards.

### Definition of prevalent multimorbidity

The diagnostic indices of the REP were searched electronically to identify the International Classification of Diseases, Ninth & Tenth Revision (ICD-9, ICD-10) codes associated with any health care visit (inpatient or outpatient) from April 1, 2010 through March 31, 2015 (5-year capture frame). These ICD-9/ICD-10 diagnostic codes from all providers indexed in the REP were used to define the 20 chronic conditions identified by the United States Department of Health and Human Services for studying multimorbidity (hypertension, hyperlipidemia, diabetes, coronary artery disease, congestive heart failure, cardiac arrhythmias, stroke, asthma, chronic obstructive pulmonary disease, arthritis, osteoporosis, chronic kidney disease, cancer, autism spectrum disorder, hepatitis, human immunodeficiency virus, depression, dementia, schizophrenia, and substance abuse disorders) [15, 16]. These conditions were selected by the Department of Health and Human Services because they are chronic (lasting at least 1 year and requiring medical attention) and potentially amenable to public health or clinical interventions [15]. The codes used to define the conditions are presented in Additional file 1: Table S1. In addition, we added anxiety to the list of chronic conditions because it is common in the United States population, for a total of 21 chronic conditions. To reduce the likelihood of false-positive diagnoses (including coding errors as well as suspect or rule-out diagnoses), we required 2 occurrences of a code (either the same code or 2 different codes within the disease code set) separated by more than 30 days. Multimorbidity was defined as having ≥2 of the 21 chronic conditions, and severe multimorbidity was defined as having ≥5 of the 21 chronic conditions.

### Calculation of the area deprivation index (ADI)

Each person’s geolocation (latitude and longitude) was calculated by linking addresses to the TIGER/Line address range shapefile provided by the United States Census [17]. Of the 206,849 adults in our cohort, 198,941 (96.2%) were successfully geocoded and retained for analysis. Geocoded data were spatially linked to census block group allowing the linkage of individual patient records with publicly available data at the census block group level (*n* = 251 census block groups). For each census block group, we calculated an area deprivation index (ADI), which is a composite measure of neighborhood socioeconomic disadvantage that uses 17 census measures capturing education, employment, income, poverty, and housing characteristics [18]. To calculate the ADI, we used 5-year estimates (2011–2015) from the American Community Survey, except in the following two instances. First, we used the 5-year estimate from 2010 to 2014 for family income. Second, we used the 2010 Census to estimate the percentage of households with a single parent with dependent(s) under age 18. The variables used in the ADI and its construction have been reported elsewhere [18–20]. Briefly, to calculate the ADI, each factor was multiplied by a factor score coefficient and summed. Factor scores were originally developed by Singh using 1990 census data [18]; however, we used updated factor scores derived from the 2000 census, as described by Knighton et al. [20]. The final score was standardized to a base mean of 100 and a standard deviation of 20. Missing values for variables in the score were imputed using the random forest imputation method, which is a nonparametric imputation method that can model highly complex missing patterns [21]. The variables with missing data were median monthly mortgage (5% missing), median gross rent (15% missing), and median home value (2% missing). For analysis, the distribution of ADI values was stratified into quintiles.

### Statistical analysis

All analyses were performed using ArcGIS 10.3 (geocoding and geographic linkage), SAS 9.4 (data cleaning and management), and R version 3.2.3 (statistical analysis and figure creation). Descriptive results for individual-level data were summarized with univariate statistics (n and %). Census and American Community Survey data were summarized as proportions for count variables and medians (Q1, Q3) for continuous variables aggregated to the block group level. Prevalences of multimorbidity were summarized at the individual and area (block group) levels. Area-level multimorbidity prevalences were estimated by dividing the number of individuals with multimorbidity in each block group by the study population residing in that block group, and were directly standardized by age and sex to the total United States 2010 Decennial Census. The area-level prevalences were plotted on a map to visualize differences in prevalence of multimorbidity and severe multimorbidity across census block groups.

Hierarchical logistic regression [22, 23], which accounted for the clustering at the census block group level, [24] was used to calculate odds ratios (OR) of multimorbidity for the highest vs. lowest quintile of each individual measure in the ADI. An unadjusted model, a model with adjustment for age (20–39, 40–49, 50–59, 60–69, 70–79, ≥80 years), sex, race (White, Black, Asian, other/unknown), and ethnicity (Hispanic, non-Hispanic), and a fully-adjusted model with further adjustment for individual level of education (high school or less, some college, college or advanced degree, unknown) were run [24, 25]. Hierarchical logistic regression was also used to model the association of the composite ADI (quintiles; with quintile 1 serving as the reference group) with multimorbidity. Unadjusted and multivariable adjusted models (as defined above) were run. The models were repeated for severe multimorbidity (≥5 chronic conditions). In addition, we tested 2-way interactions between ADI and age, between ADI and sex, and between ADI and individual level of education. Forest plots were used to display the fully-adjusted ORs in graphical form in strata by age (for the age by ADI interaction), by sex (for the sex by ADI interaction), and by education (for the education by ADI interaction).

Finally, we conducted a sensitivity analysis to assess the adequacy of our model assumptions. We measured the impact of our random forest imputation on the distribution of the ADI. The median (Q1, Q3) ADI was 102 (91, 114) before and 98 (87, 110) after imputing missing values. Because the distributions were similar, the results were presented after imputing the missing values in the ADI.

## Results

**Fig. 1:**
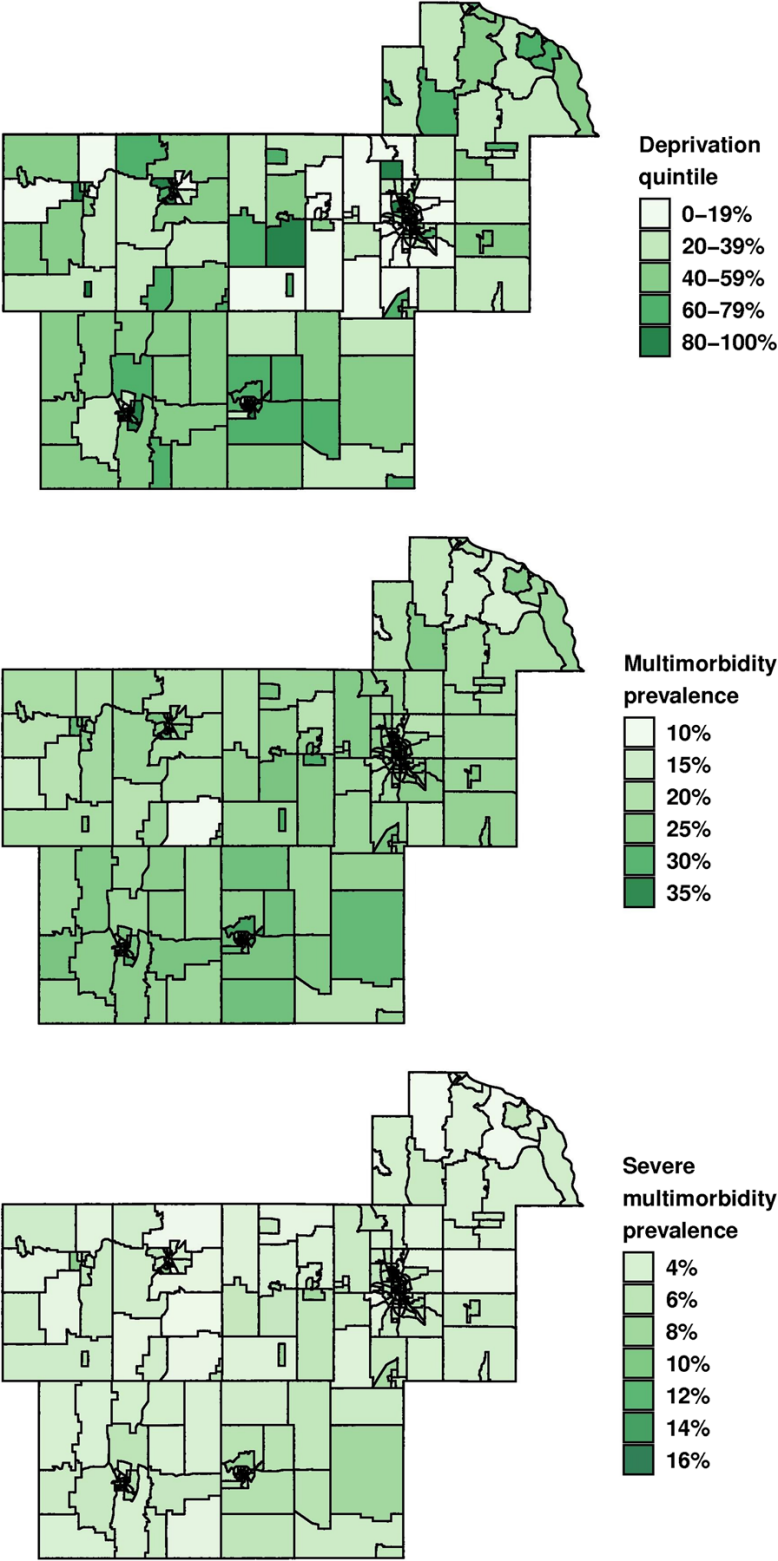
Map of area deprivation index and prevalence of multimorbidity at the block group level Top panel: Distribution of area deprivation index in quintiles; Middle panel: Prevalence of multimorbidity (≥2 chronic conditions); Bottom panel: Prevalence of severe multimorbidity (≥5 chronic conditions). The maps were plotted using the ggplot package in R. Darker colors on the map indicate greater area deprivation or higher prevalence of multimorbidity.

Among the 198,941 individuals residing in our 7-county capture region on April 1, 2015, 46.7% were male, 30.6% were aged 60 and older, and 87.7% were of White race (Table 1). Nearly 40% had multimorbidity (≥2 chronic conditions) and 10% had severe multimorbidity (≥5 chronic conditions). There were 251 census block groups in the 7-county region; each census block group had a minimum of 109 residents, a median of 707 residents, and a maximum of 2520 residents. The ADI was calculated for each of the 251 census block groups, categorized according to quintiles, and plotted on a map to show the variation of the ADI across the 7-county region (Fig. 1). Similarly, the age- and sex-standardized prevalence of multimorbidity and severe multimorbidity were plotted for each of the census block groups (Fig. 1). The median (Q1, Q3) prevalence of multimorbidity (after age- and sex-standardization to the United States 2010 population) was 23.4% (21.3%, 25.9%) and the median (Q1, Q3) prevalence of severe multimorbidity was 4.8% (4.0%, 5.7%).

**Table 1:** Descriptive characteristics of study population (*N* = 198,941)

	*N* (%)
Age, years	
20–39	69,829 (35.1)
40–49	30,698 (15.4)
50–59	37,457 (18.8)
60–69	29,111 (14.6)
70–79	18,099 (9.1)
≥ 80	13,747 (6.9)
Sex	
Female	105,948 (53.3)
Male	92,993 (46.7)
Race	
White	174,473 (87.7)
Black	6769 (3.4)
Asian	6136 (3.1)
Other/Unknown	11,563 (5.8)
Ethnicity	
Hispanic	10,683 (5.4)
Non-Hispanic	188,258 (94.6)
Individual level of education	
High school or less	41,882 (21.1)
Some college	70,069 (35.2)
College or advanced degree	27,704 (13.9)
Unknown	59,286 (29.8)
Multimorbidity (≥2 conditions)	78,527 (39.5)
Severe multimorbidity (≥5 conditions)	20,404 (10.3)

The median (Q1, Q3) of each of the 17 variables used to construct the ADI are presented in Additional file 1: Table S2. After adjusting for age, sex, race, ethnicity, and individual level of education, all of the variables in the ADI were associated with multimorbidity, with the exception of households without a telephone and households with > 1 person per room (Additional file 1: Table S3). The risk of both multimorbidity and severe multimorbidity increased with increasing ADI (Table 2). Compared with persons in the lowest quintile of ADI, those in the highest quintile had a 50% increased risk of multimorbidity (OR 1.50, 95% CI 1.39–1.62) after adjustment for age, sex, race, and ethnicity. In addition, persons in the highest quintile of ADI had more than a 60% increased risk of severe multimorbidity (OR 1.67, 95% CI 1.51–1.86). After further adjustment for individual level of education, stronger associations were observed (Table 2). Compared with persons in the lowest quintile of ADI, those in the highest quintile had a nearly 80% increased risk of multimorbidity (OR 1.78, 95% CI 1.62–1.96) and a nearly 2-fold increased risk of severe multimorbidity (OR 1.92, 95% CI 1.72–2.13).

**Table 2:** Odds ratio (95% confidence interval) of multimorbidity for quintiles of area deprivation index

Area Deprivation Index Quintile	Unadjusted odds ratio (95% CI)	P-trend	Adjusted* odds ratio (95% CI)	*P*-trend	Adjusted^†^ odds ratio (95% CI)	*P*-trend
Multimorbidity (≥2 chronic conditions)						
Quintile 1 (0–19%)	1.00 (ref)	< 0.001	1.00 (ref)	< 0.001	1.00 (ref)	< 0.001
Quintile 2 (20–39%)	1.10 (0.98–1.24)		1.03 (0.95–1.12)		1.11 (1.01–1.23)	
Quintile 3 (40–59%)	1.15 (1.03–1.29)		1.11 (1.03–1.20)		1.27 (1.15–1.39)	
Quintile 4 (60–79%)	1.23 (1.10–1.38)		1.24 (1.15–1.34)		1.45 (1.32–1.60)	
Quintile 5 (80–100%)	1.21 (1.08–1.36)		1.50 (1.39–1.62)		1.78 (1.62–1.96)	
Severe multimorbidity (≥5 chronic conditions)						
Quintile 1 (0–19%)	1.00 (ref)	< 0.001	1.00 (ref)	< 0.001	1.00 (ref)	< 0.001
Quintile 2 (20–39%)	1.14 (0.97–1.34)		1.00 (0.90–1.12)		1.05 (0.94–1.18)	
Quintile 3 (40–59%)	1.12 (0.96–1.31)		1.01 (0.91–1.12)		1.11 (1.00–1.24)	
Quintile 4 (60–79%)	1.34 (1.15–1.56)		1.21 (1.09–1.34)		1.36 (1.22–1.52)	
Quintile 5 (80–100%)	1.48 (1.27–1.72)		1.67 (1.51–1.86)		1.92 (1.72–2.13)	

*Adjusted for age, sex, race, and ethnicity

^†^Adjusted for age, sex, race, ethnicity, and individual level of education

*CI* confidence interval

**Fig. 2:**
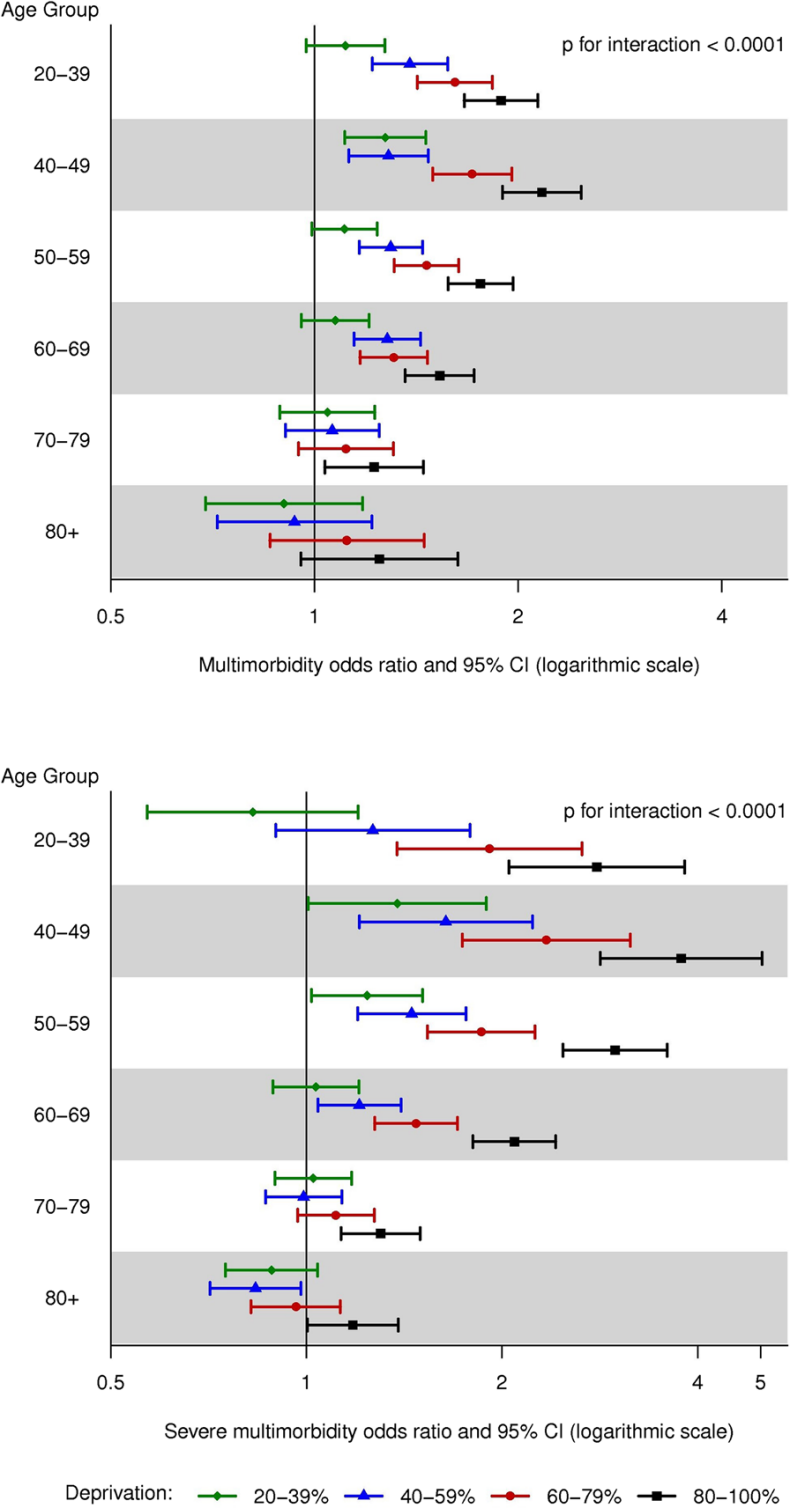
Forest plots showing the risk of multimorbidity for quintiles of area deprivation index (ADI) by age groups. Top panel: Odds ratio of multimorbidity (≥2 chronic conditions) by age groups; Bottom panel: Odds ratio of severe multimorbidity (≥5 chronic conditions) by age groups. CI, confidence interval. Odds ratios are adjusted for sex, race, ethnicity, and individual level of education. Quintile 1 (0–19%) served as the reference.

**Fig. 3:**
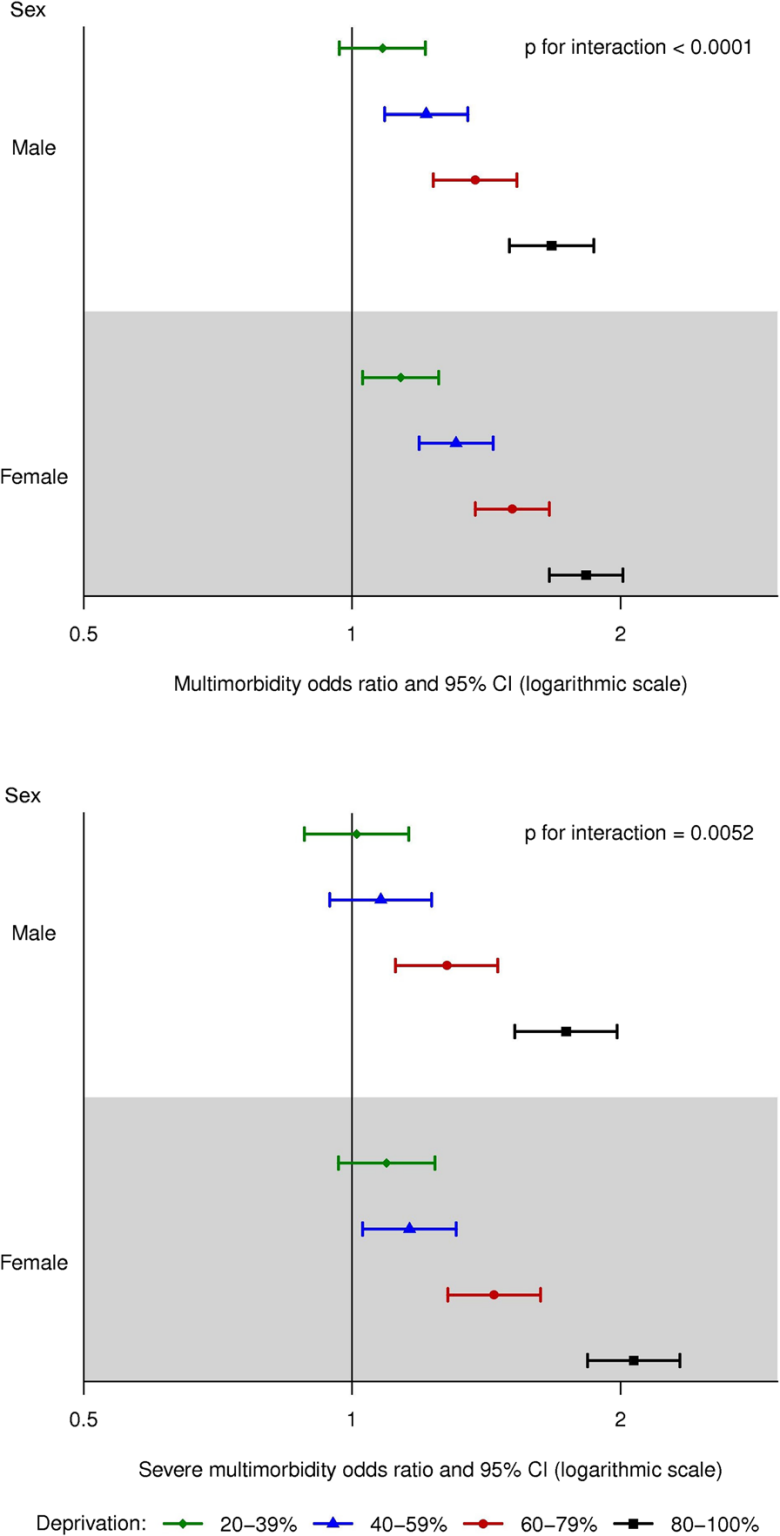
Forest plots showing the risk of multimorbidity for quintiles of area deprivation index (ADI) by sex. Top panel: Odds ratio of multimorbidity (≥2 chronic conditions) by sex; Bottom panel: Odds ratio of severe multimorbidity (≥5 chronic conditions) by sex. CI, confidence interval. Odds ratios are adjusted for age, race, ethnicity, and individual level of education. Quintile 1 (0–19%) served as the reference.

**Fig. 4:**
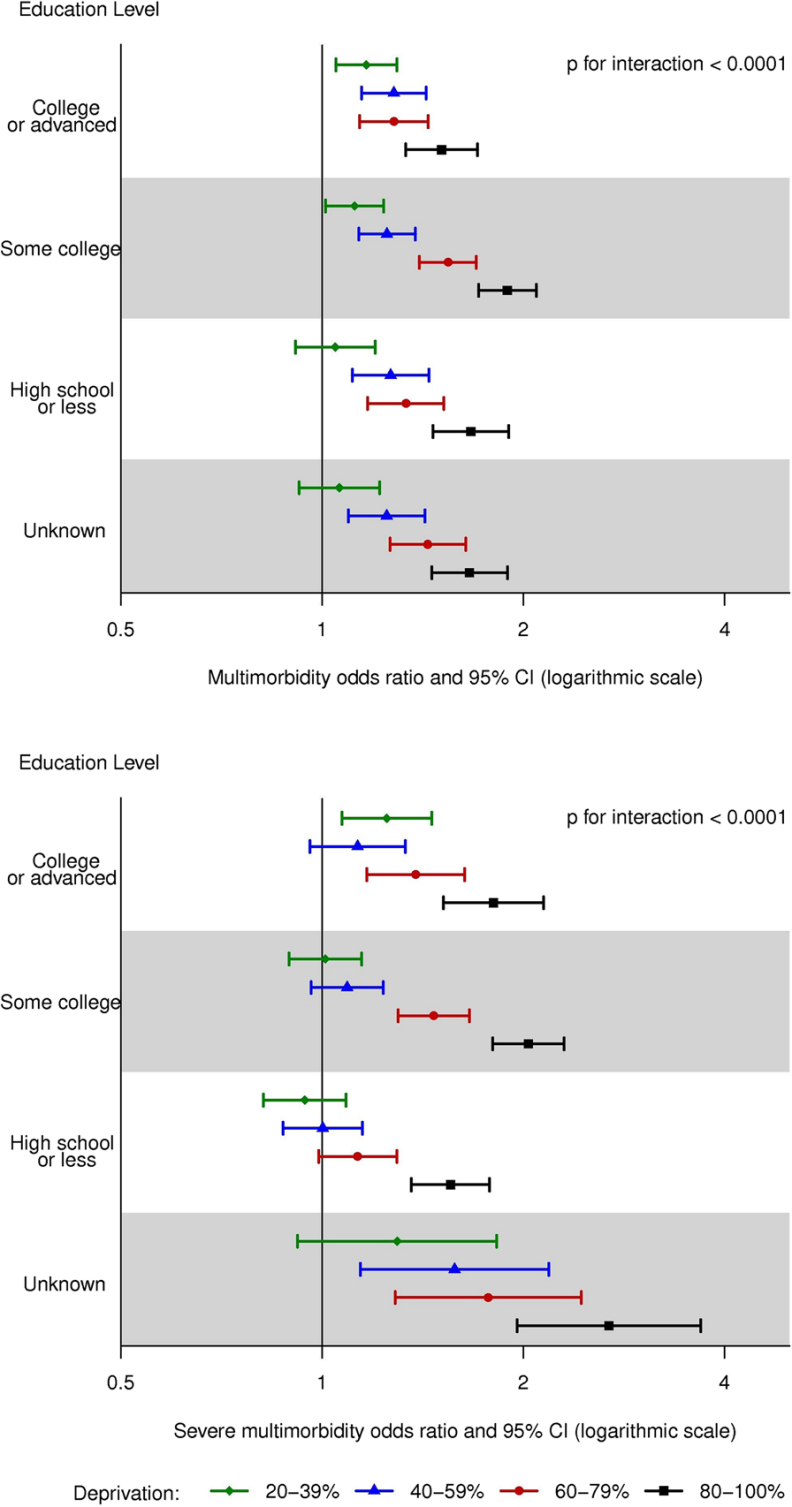
Forest plots showing the risk of multimorbidity for quintiles of area deprivation index (ADI) by individual level of education. Top panel: Odds ratio of multimorbidity (≥2 chronic conditions) by sex; Bottom panel: Odds ratio of severe multimorbidity (≥5 chronic conditions) by sex. CI, confidence interval. Odds ratios are adjusted for age, sex, race, and ethnicity. Quintile 1 (0–19%) served as the reference

There was evidence of an interaction between age and ADI in their association with multimorbidity (*p* < 0.001 for both multimorbidity and severe multimorbidity; Fig. 2). Prior to age 70, the risk of multimorbidity increased with increasing quintile of ADI, whereas after age 70, no difference in the risk of multimorbidity was observed with increasing quintiles of ADI. A similar but more extreme pattern of age with ADI interaction was observed for severe multimorbidity. In addition, an interaction between sex and ADI was observed (*p* < 0.001 for multimorbidity and *p* = 0.005 for severe multimorbidity; Fig. 3). The patterns in men and women were generally similar, with modestly stronger associations of ADI quintile with multimorbidity in women than men. Finally, an interaction between individual level of education and ADI was observed (p < 0.001 for both multimorbidity and severe multimorbidity; Fig. 4). The patterns of increasing risk of multimorbidity with increasing ADI were observed for all levels of education; however, there was less variability across quintiles of ADI for the most highly educated group (those with a college degree or an advanced degree).

## Discussion

For this study in a 7-county region in Southern Minnesota, we utilized publically available data on 17 census measures to calculate an area-level measure of socioeconomic status, the ADI. Persons residing in areas with the highest deprivation were most likely to have both multimorbidity (≥2 chronic conditions) and severe multimorbidity (≥5 chronic conditions), and associations were strengthened after adjustment for individual level of education. Furthermore, differences were observed by age group, by sex, and by individual level of education. An increasing risk of multimorbidity was observed with increasing deprivation for persons under the age of 70 years. However, no difference in risk of multimorbidity across deprivation quintile was observed among persons aged 70 years of age and older. Furthermore, the pattern of increasing multimorbidity with increasing ADI was more pronounced in women than in men. Finally, there was attenuation of the risk with less variability across quintiles of ADI for the most highly educated group.

Individual measures of socioeconomic status, including education and income, have been consistently associated with multimorbidity [3, 7, 8]. However, an understanding of the impact of the neighborhood in which one lives on multimorbidity is less well established. The communities in which people live affect the healthcare they receive and their overall health [26]. In particular, neighborhood context can affect safety, access to food, health behaviors, education, social connections, and stress [27]. Although neighborhood residence is often linked to socioeconomic status, there is also evidence of independent associations of neighborhood context with health outcomes. For example, a social experiment of nearly 4500 women with children living in public housing in high-poverty census tracts from Baltimore, Boston, New York, Chicago, and Los Angeles was conducted to assess the long-term health effects of moving to areas with low poverty [13]. Women were randomly assigned to receive housing vouchers redeemable only if they moved to a low-poverty census tract, unrestricted housing vouchers, or a reference group which did not receive vouchers. After 10 years of follow-up, the women who moved from a neighborhood with high poverty to a neighborhood with low poverty experienced reduced rates of obesity and diabetes compared to the reference group, whereas no differences in obesity or diabetes were observed between the women receiving unrestricted housing vouchers and the reference group [13]. Thus, neighborhood disadvantage itself may contribute to multimorbidity, and evidence from our study confirms this. In particular, we observed stronger associations of ADI with multimorbidity after adjustment for individual level of education. These findings indicate that the ADI is independently associated with multimorbidity, and that neighborhood disadvantage can affect health above and beyond individual measures of socioeconomic status.

Our results are consistent with previous studies conducted in Europe and Australia. Several studies in Scotland, one in England, and one in Australia reported that persons in the most deprived areas had higher rates of multimorbidity [9, 28–31], more psychological distress [29, 31], and were 2- to 3-fold more likely to have mixed physical and mental multimorbidity than persons in the least deprived areas [10]. In addition, persons living in the most deprived areas developed multimorbidity 10–15 years earlier than those residing in the most affluent areas [32]. Two additional studies from Europe reported associations of deprivation with multimorbidity by age group and sex [12, 33]. In both studies, the prevalence of multimorbidity increased with increasing deprivation in each age group, although differences between deprivation quintile were attenuated in the oldest age groups (80 years of age and older) [12, 33]. Although both sexes exhibited similar patterns of increasing multimorbidity prevalence with increasing deprivation, the associations were more marked in women in both studies [12, 33]. In our United States population, we observed an increasing risk of multimorbidity with increasing deprivation up to 70 years of age, but no difference in risk in persons aged 70 years of age and older. Furthermore, we observed generally similar patterns of deprivation with multimorbidity in men and women, but more pronounced associations in women than men.

To our knowledge, the present study is the second study conducted in the United States linking an area-level measure of socioeconomic status with multimorbidity. The first study was conducted in Olmsted County, Minnesota, and the investigators used housing characteristics to create a novel measure of socioeconomic status (HOUSES index) [11]. In that first study, low socioeconomic status based on housing characteristics was associated with increased prevalence of multimorbidity [11]. Interestingly, Olmsted County is 1 of the 7 counties included in the present study; therefore, the populations overlap to some degree. Our study, which included residents of 7 counties in Southern Minnesota, utilized an area deprivation index that incorporates 17 measures capturing education, employment, income, poverty, and housing characteristics, which may more thoroughly capture neighborhood disadvantage than housing characteristics alone. In addition, we not only reported overall associations of area deprivation with multimorbidity, but further tested interactions with age, sex, and individual level of education.

### Implications

Disparities in the prevalence of multimorbidity are apparent in relation to the neighborhood in which a person lives. Thus, neighborhood context should be considered when identifying persons who are at risk of poor health outcomes and who are eligible for interventions. Importantly, we observed that the magnitude of association varies by age, with no difference in the risk of multimorbidity across ADI quintiles in persons over age 70. We also observed that women are more affected by high ADI than men. Thus, interventions to either prevent the onset of multimorbidity or to manage chronic conditions should be targeted at younger persons, and in particular at younger women. For example, when used in conjunction with other clinical variables, the ADI may help identify patients who are at high risk for developing chronic conditions or who may be non-adherent to prescribed medication for treatment of a chronic condition. Intermountain Healthcare has demonstrated the utility of incorporating ADI for tailoring patient-centered care; the addition of ADI to an existing risk score improved the ability to identify high-risk patients that may benefit from community-based care management interventions [20]. Nevertheless, future research is warranted to better understand which socioeconomic factors are most strongly associated with multimorbidity. There are many different socioeconomic status variables that measure different aspects of individual circumstances and environmental characteristics. In our study, we were able to capture individual education level and show that associations were strengthened after adjustment for education. These findings suggest that neighborhood context is independently associated with multimorbidity, and add to the growing evidence that neighborhood context may influence health above and beyond individual measures of socioeconomic status. However, we were unable to capture other measures of socioeconomic status at an individual level, such as income. Thus, future research comparing the predictive ability of individual measures of socioeconomic status with a proxy measure of neighborhood deprivation will help inform the design of intervention approaches aimed at preventing or reducing the development of multimorbidity.

### Limitations and strengths

Some limitations should be acknowledged. First, we utilized publically available data at the census block group level which assigned the same ADI to all persons residing within a given census block group, and some misclassification may have occurred. Second, some missing variables in the ADI were imputed; however, the pre- and post-imputation distributions of ADI were similar, suggesting that the imputation increased our statistical power but did not materially affect our results. Third, we required 2 occurrences of a code separated by more than 30 days to define each chronic condition. This rule was employed to reduce false positive diagnoses, but may have resulted in some misclassification. However, we expect that any misclassification would not differ systematically across census block groups and thus would not affect our estimates. Fourth, we defined multimorbidity as a count of chronic conditions utilizing a list of 21 chronic conditions. Results may have differed if a different number or a different set of chronic conditions was used. In particular, the list of chronic conditions did not include some geriatric conditions which may have been more common in older persons, and could have affected our conclusions related to our observed differences in associations by age. Finally, the demographic and ethnic characteristics of persons captured in the E-REP are similar to those of persons living in the Upper Midwest region of the US (detailed comparisons are reported elsewhere) [14]. However, our results may not be generalizable to other areas of the country with more racial and ethnic diversity.

Some strengths should be noted. First, our large population-based study included data from nearly 200,000 persons residing in a 7-county region, including adults of all ages and without restriction on sex, insurance status, or other characteristics. Second, although we used an area-level measure of socioeconomic status, we were able to link this area measure with individual data on chronic conditions from the medical records-linkage system of the E-REP. Third, because of the large sample size, we were able to test for interactions between ADI and age, which showed that ADI was more strongly associated with multimorbidity at younger ages. Finally, we observed a stronger effect of ADI in women compared to men.

## Conclusions

In conclusion, higher ADI was associated with increased risk of both multimorbidity (≥2 chronic conditions) and severe multimorbidity (≥5 chronic conditions) after adjustment for age, sex, race, and ethnicity. Associations were strengthened after adjustment for individual level of education, suggesting that neighborhood context is independently associated with multimorbidity and may play an important role in health outcomes above and beyond individual measures of socioeconomic status. In addition, more pronounced associations were observed for younger persons and in women. Our findings underscore the importance of identifying socioeconomic disparities and implementing early interventions to prevent chronic conditions in younger persons, and in particular in younger women.

## Supplementary information

**Additional file 1: Table S1.** Diagnostic codes used to define the chronic conditions **Table S2.** Distribution of American Community Survey questions at the census block group level (*N* = 251 block groups) **Table S3.** Odds ratio (95% confidence interval) of multimorbidity for quintile 5 vs. quintile 1 of each American Community Survey question

## Data Availability

The datasets used and/or analysed during the current study are available from the corresponding author on reasonable request.

## Abbreviations

ADI: Area deprivation index
CI: Confidence interval
E-REP: Expanded Rochester Epidemiology Project
OR: Odds ratio
