# Correction to: Neighborhood socioeconomic disadvantage is associated with multimorbidity in a geographically-defined community

**DOI:** 10.1186/s12889-020-09527-2

**Published:** 2020-09-16

**Authors:** Alanna M. Chamberlain, Lila J. Finney Rutten, Patrick M. Wilson, Chun Fan, Cynthia M. Boyd, Debra J. Jacobson, Walter A. Rocca, Jennifer L. St Sauver

**Affiliations:** 1 Department of Health Sciences Research, Mayo Clinic, 200 First Street SW, Rochester, MN 55905 USA; 2 Robert D. and Patricia E. Kern Center for the Science of Health Care Delivery, Mayo Clinic, 200 First Street SW, Rochester, MN 55905 USA; 3 Division of Geriatric Medicine and Gerontology, Johns Hopkins University, 5505 Eastern Avenue, Baltimore, MD 21224 USA; 4 Department of Neurology, Mayo Clinic, 200 First Street SW, Rochester, MN 55905 USA

**Correction to: BMC Public Health 20, 13 (2020)**

**https://doi.org/10.1186/s12889-019-8123-0**

It was highlighted that in the original article [1] references 24 and 25 were erroneously cited in the second paragraph of the section Statistical analysis. This Correction article shows the corrected sentences of the paragraph.

**Statistical analysis**

Hierarchical logistic regression [22, 23], which accounted for the clustering at the census block group level, was used to calculate odds ratios (OR) of multimorbidity for the highest vs. lowest quintile of each individual measure in the ADI. An unadjusted model, a model with adjustment for age (20–39, 40–49, 50–59, 60–69, 70–79, ≥80 years), sex, race (White, Black, Asian, other/unknown), and ethnicity (Hispanic, non-Hispanic), and a fully-adjusted model with further adjustment for individual level of education (high school or less, some college, college or advanced degree, unknown) were run. Hierarchical logistic regression was also used to model the association of the composite ADI (quintiles; with quintile 1 serving as the reference group) with multimorbidity. Unadjusted and multivariable adjusted models (as defined above) were run. The models were repeated for severe multimorbidity (≥5 chronic conditions). In addition, we tested 2-way interactions between ADI and age, between ADI and sex, and between ADI and individual level of education. Forest plots were used to display the fully-adjusted ORs in graphical form in strata by age (for the age by ADI interaction), by sex (for the sex by ADI interaction), and by education (for the education by ADI interaction).
