# Supplementary material for: Neighborhood socioeconomic disadvantage is associated with multimorbidity in a geographically-defined community
Source: BMC Public Health. 2020 Jan 6;20:13. doi: 10.1186/s12889-019-8123-0 (PMC6945427; doi:10.1186/s12889-019-8123-0)
Supplement: Supplementary file 1 — Additional file 1: Table S1. Diagnostic codes used to define the chronic conditions Table S2. Distribution of American Community Survey questions at the census block group level (N = 251 block groups) Table S3. Odds ratio (95% confidence interval) of multimorbidity for quintile 5 vs. quintile 1 of each American Community Survey question [file 12889_2019_8123_MOESM1_ESM.docx]

**Supplemental Tables**

**Table S1.** Diagnostic codes used to define the chronic conditions

| **Chronic Condition** | **CCS Category^*^** | **CMS (ICD-9 Codes)^†^** |
| --- | --- | --- |
| Hypertension | 98, 99 | 401.0, 401.1, 401.9, 402.00, 402.01, 402.10, 402.11, 402.90, 402.91, 403.00, 403.01, 403.10, 403.11, 403.90, 403.91, 404.00, 404.01, 404.02, 404.03, 404.10, 404.11, 404.12, 404.13, 404.90, 404.91, 404.92, 404.93, 405.01, 405.09, 405.11, 405.19, 405.91, 405.99, 362.11, 437.2 |
| Congestive heart failure | 108 | 398.91, 402.01, 402.11, 402.91, 404.01, 404.11, 404.91, 404.03, 404.13, 404.93, 428.0, 428.1, 428.20, 428.21, 428.22, 428.23, 428.30, 428.31, 428.32, 428.33, 428.40, 428.41, 428.42, 428.43, 428.9 |
| Coronary artery disease | 100, 101 | 410.00, 410.01, 410.02, 410.10, 410.11, 410.12, 410.20, 410.21, 410.22, 410.30, 410.31, 410.32, 410.40, 410.41, 410.42, 410.50, 410.51, 410.52, 410.60, 410.61, 410.62, 410.70, 410.71, 410.72, 410.80, 410.81, 410.82, 410.90, 410.91, 410.92, 411.0, 411.1, 411.81, 411.89, 412, 413.0, 413.1, 413.9, 414.00, 414.01, 414.02, 414.03, 414.04, 414.05, 414.06, 414.07, 414.12, 414.2, 414.3, 414.8, 414.9 |
| Cardiac arrhythmias | 105, 106 | 427.31 |
| Hyperlipidemia | 53 | 272.0, 272.1, 272.2, 272.3, 272.4 |
| Stroke | 109–112 | 430, 431, 433.01, 433.11, 433.21, 433.31, 433.81, 433.91, 434.00, 434.01,434.10, 434.11, 434.90, 434.91, 435.0, 435.1, 435.3, 435.8, 435.9, 436, 997.02 |
| Arthritis | 202, 203 | 714.0, 714.1, 714.2, 714.30, 714.31, 714.32, 714.33, 715.00, 715.04, 715.09, 715.10, 715.11, 715.12, 715.13, 715.14, 715.15, 715.16, 715.17, 715.18, 715.20, 715.21, 715.22, 715.23, 715.24, 715.25, 715.26, 715.27, 715.28, 715.30, 715.31, 715.32, 715.33, 715.34, 715.35, 715.36, 715.37, 715.38, 715.80, 715.89, 715.90, 715.91, 715.92, 715.93, 715.94, 715.95, 715.96, 715.97, 715.98, 720.0, 721.0, 721.1, 721.2, 721.3, 721.90, 721.91 |
| Asthma | 128 | 493.00, 493.01, 493.02, 493.10, 493.11, 493.12, 493.20, 493.21, 493.22, 493.81, 493.82, 493.90, 493.91, 493.92 |
| Autism spectrum disorder | ICD-9 codes^§^ 299.00, 299.01 | Not applicable |
| Cancer | 11–43 | Breast cancer: 174.0, 174.1, 174.2, 174.3, 174.4, 174.5, 174.6, 174.8, 174.9, 175.0, 175.9, 233.0, V10.3. Colorectal cancer: 154.0, 154.1, 153.0, 153.1, 153.2, 153.3, 153.4, 153.5, 153.6, 153.7, 153.8, 153.9, 230.3, 230.4, V10.05. Prostate cancer: 185, 233.4, V10.46. Lung cancer: 162.2, 162.3, 162.4, 162.5, 162.8, 162.9, 231.2, V10.11. |
| Chronic kidney disease | 158 | 016.00, 016.01, 016.02, 016.03, 016.04, 016.05, 016.06, 095.4, 189.0, 189.9, 223.0, 236.91, 249.40, 249.41, 250.40, 250.41, 250.42, 250.43, 271.4, 274.10, 283.11, 403.01, 403.11, 403.91, 404.02, 404.03, 404.12, 404.13, 404.92, 404.93, 440.1, 442.1, 572.4, 580.0, 580.4, 580.81, 580.89, 580.9, 581.0, 581.1, 581.2, 581.3, 581.81, 581.89, 581.9, 582.0, 582.1, 582.2, 582.4, 582.81, 582.89, 582.9, 583.0, 583.1, 583.2, 583.4, 583.6, 583.7, 583.81, 583.89, 583.9, 584.5, 584.6, 584.7, 584.8, 584.9, 585.1, 585.2, 585.3, 585.4, 585.5, 585.6, 585.9, 586, 587, 588.0, 588.1, 588.81, 588.89, 588.9, 591, 753.12, 753.13, 753.14, 753.15, 753.16, 753.17, 753.19, 753.20, 753.21, 753.22, 753.23, 753.29, 794.4 |
| Chronic obstructive pulmonary disease | 127 | 490, 491.0, 491.1, 491.20, 491.21, 491.22, 491.8, 491.9, 492.0, 492.8, 494.0, 494.1, 496 |
| Dementia (including Alzheimer’s and other senile dementias) | 653 | 331.0, 331.11, 331.19, 331.2, 331.7, 290.0, 290.10, 290.11, 290.12, 290.13, 290.20, 290.21, 290.3, 290.40, 290.41, 290.42, 290.43, 294.0, 294.10, 294.11, 294.8, 797 |
| Depression | 657 | 296.20, 296.21, 296.22, 296.23, 296.24, 296.25, 296.26, 296.30, 296.31, 296.32, 296.33, 296.34, 296.35, 296.36, 296.51, 296.52, 296.53, 296.54, 296.55, 296.56, 296.60, 296.61, 296.62, 296.63, 296.64, 296.65, 296.66, 296.89, 298.0, 300.4, 309.1, 311 |
| Diabetes | 49, 50 | 249.00, 249.01, 249.10, 249.11, 249.20, 249.21, 249.30, 249.31, 249.40, 249.41, 249.50, 249.51, 249.60, 249.61, 249.70, 249.71, 249.80, 249.81, 249.90, 249.91, 250.00, 250.01, 250.02, 250.03, 250.10, 250.11, 250.12, 250.13, 250.20, 250.21, 250.22, 250.23, 250.30, 250.31, 250.32, 250.33, 250.40, 250.41, 250.42, 250.43, 250.50, 250.51, 250.52, 250.53, 250.60, 250.61, 250.62, 250.63, 250.70, 250.71, 250.72, 250.73, 250.80, 250.81, 250.82, 250.83, 250.90, 250.91, 250.92, 250.93, 357.2, 362.01, 362.02, 362.03, 362.04, 362.05, 362.06, 366.41 |
| Hepatitis | 6 | Not applicable |
| Human immunodeficiency virus (HIV) | 5 | Not applicable |
| Osteoporosis | 206 | 733.00, 733.01, 733.02, 733.03, 733.09 |
| Schizophrenia | 659 | Not applicable |
| Substance abuse disorders  (drug and alcohol) | 660, 661 | Not applicable |
| Anxiety^‡^ | 651 | Not applicable |

Each condition is defined by having a code in either the CCS group of codes or the CMS group of codes.

^*^The Clinical Classification Software (CCS) categories are defined by the Agency for Healthcare Research and Quality. The list of ICD-9 codes corresponding to each category can be found on the following website: <https://www.hcup-us.ahrq.gov/toolssoftware/ccs/ccs.jsp>.

^†^The ICD-9 codes were defined in the Chronic Conditions Data Warehouse of the Centers for Medicare and Medicaid Services (CMS).

^§^Autism spectrum disorder values listed in the CCS column are ICD-9 codes, not CCS categories.

^‡^Anxiety disorders were not included among the 20 conditions identified by the US Department of Health and Human Services.

**Table S2.** Distribution of American Community Survey questions at the census block group level (N=251 block groups)

| **Question** | **Median** | **Q1** | **Q3** |
| --- | --- | --- | --- |
| Family income (2014) | $68,875 | $56,003 | $85,693 |
| Income disparity^*^ | 1.78 | 0.61 | 2.61 |
| Families below poverty level | 4.6% | 1.1% | 10.1% |
| Population below 150% poverty threshold | 15.7% | 9.0% | 25.8% |
| Single parent households with dependents <18^†^ | 8.5% | 5.7% | 12.0% |
| Households without a motor vehicle | 3.2% | 1.2% | 6.8% |
| Households without a telephone | 1.3% | 0 | 2.7% |
| Occupied housing units without complete plumbing | 0 | 0 | 0 |
| Owner occupied housing units | 83.0% | 66.3% | 90.2% |
| Households with >1 person per room | 0.003% | 0 | 0.02% |
| Median monthly mortgage | $1,250 | $1,095 | $1,469 |
| Median gross rent | $726 | $621 | $909 |
| Median home value | $141,500 | $115,000 | $186,000 |
| Employed person 16+ in white collar occupation | 57.8% | 50.0% | 66.1% |
| Civilian labor force unemployed (aged 16+) | 2.5% | 1.4% | 4.7% |
| Population aged 25+ with <9yr education | 2.5% | 0.7% | 0.05% |
| Population aged 25+ with at least a high school education | 93.0% | 89.3% | 96.1% |
| ^*^Income disparity was defined as a ratio using the formula log (100*(number of households with <$10,000/number of households with ≥$50,000 in income)).  ^†^The 2010 Census was used to estimate the percentage of households with a single parent with dependent(s) under age 18. | | | |

**Table S3.** Odds ratio (95% confidence interval) of multimorbidity for quintile 5 vs. quintile 1 of each American Community Survey question

| **Question** | **Unadjusted**  **odds ratio (95% CI)** | **Adjusted^*^**  **odds ratio (95% CI)** | **Adjusted^†^**  **odds ratio (95% CI)** |
| --- | --- | --- | --- |
| Family income (2014) | 0.81 (0.72-0.91) | 0.67 (0.62-0.74) | 0.56 (0.51-0.63) |
| Income disparity^§^ | 1.18 (1.06-1.32) | 1.26 (1.16-1.37) | 1.36 (1.23-1.52) |
| Families below poverty level | 0.89 (0.79-0.99) | 1.12 (1.02-1.22) | 1.27 (1.13-1.43) |
| Population below 150% poverty threshold | 1.07 (0.96-1.20) | 1.37 (1.25-1.49) | 1.51 (1.36-1.68) |
| Single parent households with dependents <18^‡^ | 0.87 (0.78-0.98) | 1.43 (1.32-1.54) | 1.50 (1.35-1.67) |
| Households without a motor vehicle | 1.27 (1.12-1.44) | 1.25 (1.13-1.38) | 1.29 (1.14-1.47) |
| Households without a telephone | 0.85 (0.70-1.03) | 1.07 (0.90-1.26) | 0.99 (0.80-1.21) |
| Occupied housing units without complete plumbing | 0.89 (0.79-1.00) | 0.85 (0.77-0.94) | 0.76 (0.67-0.87) |
| Owner occupied housing units | 0.92 (0.81-1.03) | 0.70 (0.64-0.77) | 0.66 (0.59-0.75) |
| Households with >1 person per room | 0.78 (0.66-0.92) | 1.02 (0.89-1.16) | 0.94 (0.79-1.12) |
| Median monthly mortgage | 0.78 (0.68-0.88) | 0.66 (0.60-0.72) | 0.55 (0.49-0.62) |
| Median gross rent | 0.77 (0.69-0.87) | 0.79 (0.71-0.87) | 0.64 (0.57-0.72) |
| Median home value | 0.81 (0.72-0.91) | 0.66 (0.61-0.72) | 0.53 (0.48-0.58) |
| Employed person 16+ in white collar occupation | 0.79 (0.70-0.88) | 0.73 (0.67-0.80) | 0.59 (0.53-0.66) |
| Civilian labor force unemployed (aged 16+) | 1.00 (0.88-1.13) | 1.23 (1.11-1.36) | 1.31 (1.15-1.49) |
| Population aged 25+ with <9yr education | 1.07 (0.96-1.19) | 1.10 (1.01-1.20) | 1.23 (1.10-1.38) |
| Population aged 25+ with at least a high school education | 0.84 (0.75-0.94) | 0.72 (0.67-0.79) | 0.60 (0.54-0.67) |

^*^Adjusted for age, sex, race, and ethnicity.

^†^Adjusted for age, sex, race, ethnicity, and individual level of education.

^§^Income disparity was defined as a ratio using the formula log (100*(number of households with <$10,000/number of households with ≥$50,000 in income)).

^‡^The 2010 Census was used to estimate the percentage of households with a single parent with dependent(s) under age 18.

CI, confidence interval.
